# Effect of adjunctive single high-dose vitamin D_3_ on outcome of community-acquired pneumonia in hospitalised adults: The VIDCAPS randomised controlled trial

**DOI:** 10.1038/s41598-018-32162-2

**Published:** 2018-09-14

**Authors:** Sandy Slow, Michael Epton, Malina Storer, Rennae Thiessen, Steven Lim, James Wong, Paul Chin, Pleayo Tovaranonte, John Pearson, Stephen T. Chambers, David R. Murdoch, VIDCAPS Group, David Jardine, Alan Pithie, Christopher Warren, Suzanne Faville, Avinesh Shankar, Evan Cameron, Tom Evans, Pamela Mooi, Christy McDonald, Huan Chan, John Llewelyn, Michael Liu

**Affiliations:** 1 Department of Pathology and Biomedical Science, University of Otago, Christchurch, New Zealand; 2 Canterbury Respiratory Research Group, Canterbury District Health Board, Christchurch, New Zealand; 3 Radiology Department, Christchurch Hospital, Canterbury District Health Board, Christchurch, New Zealand; 4 Department of General Medicine, Christchurch Hospital, Canterbury District Health Board, Christchurch, New Zealand; 5 Present Address: Waimairi Road Medical Centre, Christchurch, New Zealand; 6 Present Address: Clinical Pharmacology, Christchurch Hospital, Canterbury District Health Board, Christchurch, New Zealand; 7 Present Address: Rolleston Central Health, Rolleston, Canterbury, New Zealand

## Abstract

Low vitamin D status is associated with increased risk of pneumonia, greater disease severity and poorer outcome. However, no trials have examined the effect of adjunctive vitamin D therapy on outcomes in adults with community-acquired pneumonia (CAP). We conducted a randomised, double-blind, placebo-controlled trial examining the effects of adjunctive vitamin D in adults hospitalised with CAP. Participants were randomised to either a single oral dose of 200,000 IU vitamin D_3_ or placebo. The primary outcome was the complete resolution of chest radiograph infiltrate at 6 weeks post-study treatment. Secondary outcomes included length of hospital stay, intensive care admission and return to normal activity. Only participants who completed the study or died within the 6 week period were included in the analysis (n = 60 vitamin D, n = 57 placebo). Adjunctive vitamin D did not have any effect on the primary outcome (OR 0.78, 95% CI 0.31 to 1.86, p = 0.548). However, there was evidence it increased the complete resolution of pneumonia in participants with baseline vitamin D levels <25 nmol/L (OR 17.0, 95% CI 1.40–549.45, P = 0.043), but this did not reach statistical significance using exact methods (OR 13.0, 95%CI 0.7–960.4, P = 0.083). There were no significant effects for any secondary outcome.

## Introduction

Vitamin D has important roles both in innate immunity, through the production of antimicrobial peptides (cathelicidins and β-defensins), and in adaptive immunity, which includes regulating T-lymphocyte cytokine production to suppress the pro-inflammatory response to infection^1–5^. These observations have prompted investigation of the potential role of vitamin D in the prevention or management of a variety of infections, including acute infections of the respiratory tract.

A recent individual participant meta-analysis of 25 randomised controlled trials showed that vitamin D supplementation was protective against acute respiratory infections, with the most benefit observed in individuals with vitamin D deficiency and in those that received small regular doses (daily or weekly) as opposed to larger less frequent doses (monthly or quarterly)^6^. However, the potential role of vitamin D as an adjunctive treatment for acute respiratory infections is still unclear. Randomised controlled trials in children using either a single large dose^7–9^ or smaller daily doses^10^ showed no change in the resolution of acute clinical features and inconsistent results in preventing recurrence of pneumonia despite a high prevalence of vitamin D deficiency^7–10^. There are no studies reported for lower respiratory tract infections in adults except in tuberculosis where adjunctive vitamin D has been shown to hasten sputum culture conversion but only in those patients who have the vitamin D receptor *T*aqI *tt* genotype^11^.

Previously we have observed a high prevalence of vitamin D insufficiency/deficiency among hospitalised participants of an adult community-acquired pneumonia (CAP) study^12^ from our region (n = 300; median serum 25 hydroxyvitamin D (25OHD) concentrations of 28 nmol/L, unpublished data). This observation, combined with the absence of data on vitamin D adjunctive treatment in adults with CAP, prompted us to assess the effect of a single oral dose of vitamin D as adjunctive therapy on the outcome of adults hospitalised with CAP. We hypothesised that adjunctive vitamin D would be associated with improved outcomes from CAP, especially in patients with vitamin D insufficiency or deficiency.

## Methods

### Study design and participants

The study was a randomised, double-blind, placebo-controlled trial conducted in Christchurch, New Zealand. Participants were adults (aged ≥ 18 years) admitted to Christchurch Hospital during June 2013 to March 2016 with CAP, which was defined as: pneumonia that has been acquired outside of a hospital or health care setting where the patient has new inflammatory infiltrate on chest radiograph and acute illness with clinical features of pneumonia.

Potential participants were identified by clinical staff from Christchurch Hospital’s General Medicine and Respiratory Services upon admission to hospital and then screened for eligibility. Exclusion criteria at screening were admission to hospital >48 hours prior to enrolment, and an anticipated inability to attend a follow-up appointment at 6 weeks post-study treatment for blood sample collection and a chest radiograph.

Potential participants were further screened by investigators and excluded if any of the following were present: (1) pneumonia was not the principal reason for admission; (2) pneumonia associated with bronchial obstruction, bronchiectasis or known tuberculosis; (3) hospital admission in the previous two weeks such that hospital acquired pneumonia could not be ruled out; (4) use of vitamin D supplements other than as part of a daily multivitamin preparation (in which the daily intake was >400 IU); (5) use of immunosuppressants (e.g. daily prednisone use >10 mg); (6) history of hypercalcemia or nephrolithiasis; (7) sarcoidosis; (8) kidney disorders requiring dialysis or polycystic kidney disease; (9) cirrhosis; (10) baseline plasma calcium (corrected for plasma albumin concentration) >2.6 mmol/L; (11) enrolment in other research that would conflict with full participation in the study or confound the observations or interpretation of the study findings; (12) known or suspected pregnancy or breastfeeding; (13) current malignancy diagnosis in which the cancer was aggressive and prognosis was poor.

The study was approved by the Southern Health and Disability Ethics Committee (13/STH/41) and all participants gave written informed consent. The trial was conducted according to the principles of the Declaration of Helsinki and was registered with the Australia New Zealand Clinical Trials Registry, http://www.anzctr.org.au (ACTRN12613000582752) on May 24^th^, 2013.

### Randomisation and masking

Participants were assigned using computer-generated randomisation to receive treatment with either a single oral dose of 200,000 IU vitamin D_3_ or placebo. Both the vitamin D_3_ and placebo tablets were sourced from Tishcon Corp (NY, USA) and were identical in appearance. The randomisation process and bottling of tablets were performed by Christchurch Hospital Pharmacy under the direction of the study biostatistician (J.P.) to ensure those assessing eligibility (including outcome assessors) or administering the intervention were blinded to treatment allocation. Research staff witnessed administration of the single-dose treatment by hospital medical staff (nurses or attending physicians) to all participants immediately following enrolment into the study.

### Study procedures

Information on baseline characteristics was obtained by interviewer administered questionnaire at screening and included data on demographics, medical history, smoking, current medications and supplement use. Clinical data including routine physiological, haematological and biochemical information, length of stay, mortality, intensive care unit admission, antimicrobial therapy and readmission to hospital between study enrolment and the 6 week follow-up period were collected from patient hospital records. At baseline, a CURB-65 score for pneumonia severity^13^ and an Early Warning Score (EWS)^14,15^ for severity of illness was calculated for each patient from the clinical data collected.

Chest radiographs taken at both baseline and at 6 weeks post-study treatment were reviewed independently by the two study radiologists (R.T. and S.L.) and the results compared. Any discrepancy was resolved by further review and consensus. At 6 weeks post study treatment, investigators also administered a brief questionnaire over the telephone regarding the resolution of the participant’s symptoms and if they had returned to normal activity.

The primary end-point was the clearance on chest radiograph of inflammatory infiltrate and being alive at 6 weeks post-study treatment. Secondary end-points were length of hospital stay, admission to intensive care, mortality within the 6 week follow-up period, rates of relapse and readmission to hospital and the resolution of symptoms and return to normal activity.

Plasma calcium and 25-hydroxyvitamin D (25OHD) concentrations were measured at baseline and 6 weeks post study treatment. Plasma calcium was measured in real time to monitor safety (Abbott c8000 analyser, Abbott Laboratories). Baseline plasma 25OHD was measured prior to the 6 week follow-up appointment, while those collected at follow-up were stored at −80 °C and were not measured until all participants had completed the study to maintain blinding. The 25OHD concentrations were measured by liquid chromatography-tandem mass spectrometry (ABSciex API 4000).

### Statistical analysis

On the assumption that the intervention would increase the resolution of pulmonary inflammatory infiltrate on follow-up chest radiograph at 6 weeks post-study treatment from 50% to 70% of patients, we calculated that a sample size of 360 participants would be required for a power of 80% at the 0.05 level of significance. Based on our previous data the prevalence of vitamin D deficiency (plasma 25OHD <25 nmol/L) in hospitalised CAP patients in Christchurch, this number was increased to up to 400 participants to ensure we had a high proportion (approximately n = 120) of those with deficiency (<25 nmol/L) at baseline to increase the possibility of seeing an effect.

Randomised participants were divided into three groups: those that completed or died within the 6 week study period; those that were excluded as ‘ineligible on review’ because either pneumonia was not the primary diagnosis or they did not meet the radiological case definition of pneumonia following further review of the baseline chest radiograph; and those that withdrew during the 6 week study period. Only eligible participants that completed or died within the 6 week follow-up period were included in the primary analysis on a modified intention to treat basis. Dropout events were reported on and examined for differential compliance between treatment and placebo arms. Primary analysis was logistic regression of clear chest radiograph and being alive at 6 weeks post-study treatment on treatment arm with covariates for age (dichotomous at the median), gender (dichotomous), ethnicity (dichotomous; Māori/Non-Māori) and season that the participant was recruited in (spring, summer, autumn and winter). Additional analyses included covariates for initial baseline 25OHD concentrations at deficient (<25 nmol/L) and insufficient (<50 nmol/L) concentrations. All data was analysed using R (version 3.2.1 Vienna, Austria) statistical software. Testing was 2-sided with statistical significance set at p < 0.05.

## Results

### Study recruitment, follow-up and baseline characteristics

**Figure 1:**
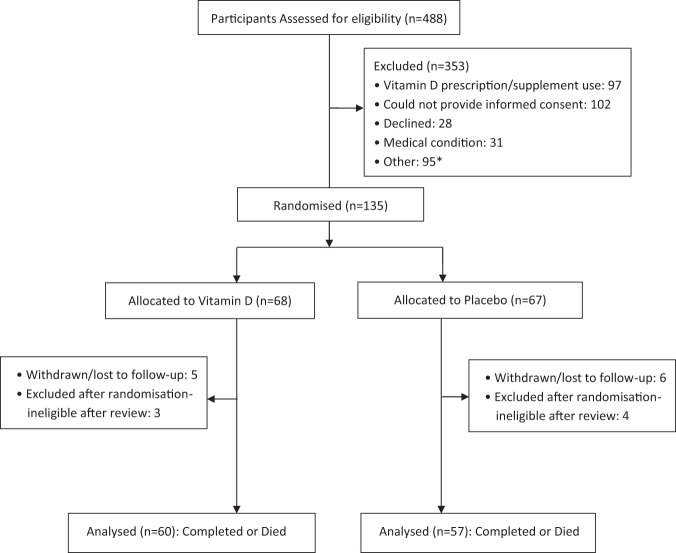
Participant Flow. *The predominant ‘Other’ reasons participants were excluded from the study were: use of immunosuppressants, previous history of nephrolithiasis and cirrhosis.

Figure 1 shows the study diagram. Of 488 potential participants screened, 135 were eligible for inclusion and were randomised to a treatment group, giving an overall recruitment rate of nearly 30% of those screened. However, most of these particpants were recruited in the first 18 months of the study as the number of potential participants that were ineligible because of prior consumption of vitamin D supplements markedly increased as the study progressed. This limitation combined with the high number of patients who could not give informed consent, either because of confusion and delerium, or because of pre-existing conditions (e.g. dementia) meant that enrolment dropped to <10% of patients screened in the last 18 months of the study and the recruitment target of up to 400 participants became impractical. The decision was made to stop enrolment at the end of March 2016.

Of the 135 participants randomised to treatment, 112 (83%) completed the study, 5 (4%) died during follow-up (3 vitamin D group; 2 placebo group), 11 (8%) withdrew or were lost to follow-up and 7 (5%) were excluded following randomisation as ‘ineligible on review’ because either subsequent medical investigations/interventions discounted CAP as the primary reason for hospital admission or they did not fulfil the radiological case defiition of pneumoniation following further radiological review of the baseline chest radiograph by the study radiologists. All participants that completed the study or died within the 6 week follow-up period (n = 117) were included in a modified intention-to-treat analysis.

Table 1 shows the baseline characteristics of the 117 participants that were included in the analysis. The mean age at recruitment was 63 years and 74 (63%) were male. In addition to receiving either vitamin D or placebo, all patients received standard antimicrobial therapy for the treatment of CAP, which at Christchurch Hospital is based on the British Thoracic Society guidelines. Briefly, those patients with CURB-65 scores of 0 or 1 received monotherapy with a beta-lactam antibiotic (amoxicillin or amoxicillin-clavulanic, or cefuroxime for those with penicillin allergies), while those with a CURB-65 score 2 or greater, were given dual therapy of a beta-lactam and macrolide (azithromycin). The study treatment groups were evenly balanced on all characteristics except for pleural effusion.

**Table 1:** Baseline Characteristics.

Characteristic	Number of Participants (%)	
	Vitamin D (n = 60)	Placebo (n = 57)
Age, years (mean ± SD)	65.0 ± 14.5	60.9 ± 17.3
Male	35 (58)	39 (68)
^a^Ethnicity		
Non-Māori	56 (93)	54 (95)
Māori	4 (7)	5 (9)
Season of Recruitment		
Spring	22 (37)	22 (39)
Summer	8 (13)	6 (11)
Autumn	15 (25)	15 (26)
Winter	15 (25)	14 (24)
Smoking Status		
Never	17 (28)	22 (39)
Current	12 (20)	7 (12)
Ex-smoker	31 (52)	28 (49)
Co-morbidities		
COPD	13 (22)	10 (18)
Asthma	13 (22)	14 (25)
Heart failure	1 (2)	3 (5)
Diabetes	10 (17)	8 (14)
Cerebrovascular disease	3 (5)	1 (2)
Renal disease	3 (5)	4 (7)
Liver disease	0	1 (2)
Immune suppression	1 (2)	0
^b^Early warning score		
0	1 (2)	1 (2)
1	8 (13)	7 (12)
2	13 (22)	15 (26)
3	14 (23)	13 (22)
4	8 (13)	5 (9)
5	4 (7)	7 (12)
≥6	11 (18)	9 (16)
CURB-65 score		
0	13 (22)	17 (30)
1	26 (43)	15 (26)
2	15 (25)	17 (30)
3	4 (7)	6 (11)
4	2 (3)	2 (3)
5	0	0
Radiology		
Lobar consolidation	46 (77)	45 (79)
Multilobar consolidation	14 (23)	12 (21)
^c^Pleural effusion	16 (27)	6 (11)
Plasma 25-hydroxyvitamin D nmol/L (mean ± SD)	47.9 ± 22.0	49.4 ± 21.6
^d^Plasma calcium mmol/L (mean ± SD)	2.4 ± 0.1	2.3 ± 0.1

^a^Participants could identify with more than one ethnic group, therefore percentages do not add to 100. One participant in the vitamin D group declined to answer.

^b^Data was not obtained for 1 patient in the vitamin D treatment group.

^c^Pleural effusion was not assessable on radiographs for 4 patients (vitamin D n = 1, placebo n = 3).

^d^Plasma calcium values stated are corrected for plasma albumin concentration.

### Plasma 25-OHD concentrations

Mean baseline plasma 25-OHD concentration was 48.7 ± 21.6 (SD) nmol/L and 15 participants (n = 9 vitamin D; n = 6 placebo) had levels below 25 nmol/L. In the vitamin D group, average plasma 25OHD concentrations at 6 weeks post study treatment increased to 99.7 ± 20.5 nmol/L and was significantly higher than the placebo treatment group (55.8 ± 24.2 nmol/L; p = 0.05).

### Clearance of pneumonia and recovery

A total of 57 participants (49%; n = 30 vitamin D; n = 27 placebo) had complete resolution of pulmonary inflammatory infiltrate on chest radiograph and were alive at 6 weeks post study treatment, with no significant difference between the two groups (Table 2). Stratifying by baseline 25OHD at 25 nmol/L showed no significant difference in either low or normal/high baseline vitamin D groups (Table 2).

**Table 2:** Outcome measures by treatment group.

Characteristic^a^	Vitamin D	Placebo	Vitamin D versus Placebo	P value
			*Risk Ratio (95% CI)*	
ICU admission	0	3 (5)	0.95 (0.89,1.01)	0.11
Days in-patient stay (mean + SD)^b^	3.8 ± 3.1	4.8 ± 6.5	−1.01 (−2.90,0.89)	0.29
Death during 6 week follow-up	3 (5)	2 (4)	1.01 (0.94, 1.10)	1
Discharged with antibiotics	53 (88)	50 (88)	0.92 (0.31, 2.68)	1
*Readmission to hospital or doctor visit during follow-up*				
Relapse of CAP	2 (3)	5 (9)	0.94 (0.86, 1.04)	0.26
Exacerbation of existing condition	0	2 (4)	0.96 (0.92, 1.01)	0.24
Other	23 (38)	20 (35)	1.04 (0.79, 1.37)	0.85
Further antibiotics during follow-up	17 (28)	18 (32)	0.95 (0.75, 1.20)	0.84
*Persisting symptoms at 6 weeks*				
Cough	18 (30)	20 (35)	0.92 (0.72, 1.19)	0.69
Chest pain	10 (17)	9 (16)	1.01 (0.86, 1.18)	1
Short of breath	24 (40)	23 (40)	0.98 (0.73, 1.32)	1
Returned to normal activity at 6 weeks	34 (57)	28 (49)	1.15 (0.78, 1.69)	0.46
Days off work (mean ± SD)^c^	14.8 ± 13.2	13.5 ± 11.1	1.31 (−6.01, 8.62)	0.72
*Chest x-ray findings at 6 weeks*				
Complete resolution	30 (50)	27 (47)	0.85 (0.39, 1.86)	0.84
Baseline 25OHD < 25 nmol/L^d^	6 (67)	1 (17)	10.00 (0.78, 128.77)	0.12
Baseline 25OHD ≥ 25 nmol/L^d^	24 (47)	26 (51)	1.11 (0.54, 2.30)	0.85
Moderate improvement	21 (35)	19 (33)	1.02 (0.78, 1.32)	1
Mild improvement	5 (8)	4 (7)	1.01 (0.91, 1.12)	1
Unchanged	0	3 (5)	0.95 (0.89, 1.01)	0.11
Progression	1 (2)	2 (4)	0.98 (0.92, 1.04)	0.61
Plasma 25-hydroxyvitamin D nmol/L at 6 weeks (mean ± SD)^e^	99.7 ± 20.5	55.8 ± 24.2	43.87 (35.15, 52.60)	<0.001
Plasma calcium mmol/L at 6 weeks (mean ± SD)^f^	2.4 ± 0.1	2.4 ± 0.1	0.03 (−0.01, 0.06)	0.1

^a^Unless otherwise stated values presented are the number of participants (%). No follow-up data was available for patients that died within the 6-week study period (vitamin D n = 3, placebo n = 2).

^b^Includes days of stay for those who died in hospital (vitamin D n = 2; placebo n = 2).

^c^Twenty five (42%) participants in the vitamin D treatment group and 23 (40%) participants in placebo treatment group were employed at the time of their admission to hospital for CAP. At 6 weeks follow-up 6 participants had not returned to work (vitamin D n = 3, placebo n = 3).

^d^(%) is calculated on the total number of participants in each treatment arm that either had plasma 25OHD levels < 25 nmol/L or ≥25 nmol/L.

^e^Data were not obtained for 13 participants (vitamin D n = 7; placebo n = 6) at 6 weeks post-treatment.

^f^Plasma calcium values stated are corrected for plasma albumin concentrations. There was missing data for 14 participants (vitamin D n = 8; placebo n = 6) at 6 weeks post-treatment.

As a result of early termination, the study produced a dataset on 117 participants, sufficiently small to risk inflationary bias in estimates from logistic regression fitted with maximum likelihood^16^. To produce unbiased estimates the same model was refitted using an approximation to exact inference^17^ which uses Markov chains to estimate parameters in the logistic model. Our model was fitted with 50,050,000 simulations, the first 50,000 were discarded as a burn in period. The minimum chain length for any parameter was 18,966, sufficiently long for stable parameter estimation, and the maximum Monte Carlo standard error for a p value was 0.006, for both treatment groups. In addition, gender was dropped from the logistic regression model as there was no evidence it had a significant effect and did not materially change the conclusions about the other variables.

Logistic regression did find that supplementation had a significantly greater effect on those with low baseline 25OHD, however using exact methods produced a lower estimate of additional risk that was not statistically significant (Table 3). This effect was not observed when the data was analysed by baseline 25OHD levels <50 nmol/L. Gender, ethnicity and season of recruitment did not contribute to the effect when included in the multivariate model. No statistically significant differences were noted for any of the secondary outcomes (Table 2). A full intention to treat analysis using all randomised participants (n = 68 vitamin D; n = 67 placebo) did not materially alter the findings.

**Table 3:** Effect of vitamin D adjunctive therapy and baseline vitamin D deficiency (<25 nmol/L) on complete resolution of CAP and being alive at 6 weeks post-treatment.

Variable	Maximum Likelihood		Exact	
	OR (95% CI)	p value	OR (95% CI)	p value
Age >65 years	0.46 (0.21, 0.98)	0.046	0.48 (0.20, 1.06)	0.059
Treatment	0.79 (0.35, 1.74)	0.552	0.78 (0.31, 1.86)	0.548
Baseline Vitamin D < 25 nmol/L	0.18 (0.01, 1.23)	0.129	0.17 (0.00, 1.83)	0.189
Treatment and Baseline Vitamin D < 25 nmol/L	16.99 (1.40, 469.45)	0.043	13.00 (0.71, 960.38)	0.083

Odds Ratio (OR) with 95% Confidence Intervals (CI) and p values from logistic regression fitted with Maximum Likelihood or Exact methods.

### Safety

Mean corrected plasma calcium levels were significantly higher at baseline for the vitamin D group, however concentrations were not significantly different between the two groups at 6 weeks post study treatment. There were no cases of asymptomatic hypercalcemia (corrected plasma calcium >2.6 mmol/L). There were 16 serious adverse events (8 in each group) and 83 other adverse events (vitamin D n = 44; placebo n = 39) recorded during the study, none of which were thought to be related to vitamin D supplementation.

## Discussion

The main finding from this study is that a single dose of 200,000 IU vitamin D in conjunction with standard antimicrobial therapy and supportive care did not improve outcomes in adults hospitalised with CAP. However, for patients with low baseline vitamin D levels (25OHD <25 nmol/L) and alive at 6 weeks post-study treatment, there was evidence that adjunctive vitamin D therapy increased the proportion with complete resolution of the radiographic manifestations of pneumonia. This is consistent with an effect of vitamin D on the regulation of the pro-inflammatory response^3–5^. However, while this finding was statistically significant with logistic regression modelling, it did not reach statistical significance using exact methods.

There has been considerable interest in the role of vitamin D for the prevention of acute respiratory infections. A recent individual participant meta-analysis showed that vitamin D supplementation does have a protective effect against acute respiratory infections, particularly in individuals with vitamin D deficiency^6^. However, there are few studies of the effect of vitamin D as adjunct treatment for pneumonia. Four randomised controlled trials have examined adjunct vitamin D therapy for pneumonia, all in children under 5 years of age^7–10,18^. Despite differences in treatment dose and primary outcome measures, all four trials showed no improvement in the resolution of the acute manifestations of pneumonia, despite high prevalences of vitamin D deficiency. However, one of these trials in hospitalised Afghani children did show a significantly reduced risk of a repeat episode of pneumonia within 90 days of supplementation^8^. Ours is the first trial of adjunct vitamin D treatment in adults with CAP.

A major limitation of our study was the failure to meet our planned sample size. Consequently, we are reporting our findings with caution given that we were unlikely to be sufficiently powered to properly assess our outcome measures. We do, however, think it is important to report our findings in order for the data to be available for meta-analyses and to stimulate further research on this topic. Our concerns about the potential for inflationary bias in estimates from logistic regression fitted with maximum likelihood prompted us to also use exact methods in order to produce unbiased estimates^17^. This resulted in a shift from statistical significance to borderline significance for the effect of vitamin D treatment on radiographic resolution in participants with low baseline vitamin D levels (25OHD <25 nmol/L). Despite the lack of confidence in the effect of vitamin D treatment in these patients, the trend is in keeping with prevention trials, which show most benefit in individuals with vitamin D deficiency^6^, and with observational studies where low vitamin D status is associated with an increased risk of pneumonia, more severe disease and poorer outcomes^19–21^. At the very least, our findings warrant further study in a larger population that includes a sizeable proportion of patients with low baseline vitamin D levels.

A further limitation of our study cohort is that it may not be truly representative of the adult CAP population. The patients that were screened but ruled ineligible were significantly older, had more severe pneumonia and were in generally poorer health with a higher rate of comorbid disease. As a consequence, the generalisability of our findings may be limited to younger adults that are less unwell. Furthermore, a single large bolus dose of vitamin D may not be the most appropriate treatment regime. Several studies have shown that smaller doses at more frequent intervals, such as daily or weekly, are more effective for the prevention of acute respiratory infections^6,22^. It is unclear if the same applies for treating an acute episode of pneumonia. The dose utilised in this study was chosen to ensure that those with frank deficiency would atttain adequate concentrations. While there is no data for the expected response to vitamin D_3_ supplementation in adults with CAP, a previous pharmacokinetic study showed that following a 100,000 IU dose of vitamin D_3_, 25OHD concentrations in healthy adults reached maximum concentrations seven days after supplementation, thereafter declining and reaching pre-supplement concentrations at around 84 days^23^. This time course appears to be consistent with our findings in that those participants who were randomised to vitamin D_3_ had higher circulating 25OHD levels 6 weeks post-study treatment compared with those at baseline. However, because vitamin D can influence the immune response through various mechanisms^1–3,5,24^, it is plausible that the optimal dosing regimen may be different to the one utilised here. It may also depend on whether a preventative or treatment effect is required, but this needs further assessment.

The strengths of the study include the use of chest X-rays to confirm pneumonia cases and to assess its resolution, and the measurement of vitamin D status at both baseline and 6 weeks post study treament. In addition, it was a randomised, well-conducted trial where the primary outcome and other clinical measures were assessed by experienced physicians. In terms of safety, no adverse events, side effects or episodes of hypercalcemia were attributed to vitamin D adjunctive therapy.

In conclusion, we report that a single 200,000 IU dose of vitamin D_3_ does not significantly improve the radiological resolution of pneumonia in hospitalised adults overall, but there is evidence to suggest it may be of benefit for those who are frankly deficient. This finding is consistent with the known role of vitamin D in regulating the pro-inflammatory response. Further research is required to confirm these findings in other populations.

## Electronic supplementary material

Consort Checklist

Study Protocol

## Data Availability

The datasets generated and analysed during the current study are available from the corresponding author on reasonable request.
