# Supplementary material for: Effect of adjunctive single high-dose vitamin D3 on outcome of community-acquired pneumonia in hospitalised adults: The VIDCAPS randomised controlled trial
Source: Sci Rep. 2018 Sep 14;8:13829. doi: 10.1038/s41598-018-32162-2 (PMC6138743; doi:10.1038/s41598-018-32162-2)
Supplement: Supplementary file 2 — Study Protocol [file 41598_2018_32162_MOESM2_ESM.docx]

**STUDY PROTOCOL**

**Effect of Adjunct High-Dose Vitamin D on Community Acquired Pneumonia in Hospitalised Adults - A Randomised Controlled Trial**

**(Vitamin D and Community Acquired Pneumonia Study [VIDCAPS])**

Funder:

University of Otago

**TABLE OF CONTENTS**

1. SYNOPSIS 4

2. BACKGROUND 5

2.1. Community Acquired Pneumonia 5

2.1.1. Epidemiology of Community Acquired Pneumonia 5

2.1.2. Clinical and Economic Importance of Community Acquired

Pneumonia 5

2.2. Overview of Vitamin D 5

2.2.1. Vitamin D metabolism 5

2.2.2. 25OHD Levels Required for Optimum Health 6

2.2.3. Vitamin D Levels and New Zealand 6

2.2.4. Vitamin D Supplementation, Dosing Regimes and Absorption 7

2.3. Vitamin D and Respiratory Infection 7

2.3.1. Vitamin D activates Cathelicidin Production and Suppresses

Inflammation in Respiratory Infection 7

2.3.2. Vitamin D and Tuberculosis 8

2.3.3. Epidemiological Studies of Rickets, Vitamin D and Respiratory

Infection 8

2.3.4. Trials of Vitamin D Supplementation and Respiratory Infection 9

2.3.5. Vitamin D as an Adjunct Therapy 10

2.4. Genetics Sub-study 11

3. RESEARCH DESIGN AND METHODS 11

3.1. Hypothesis 11

3.2. Study Overview 11

3.3. Participants 11

3.4. Intervention 12

3.5. Study Period 13

3.6 Data Collection 13

3.6.1. Chest X-Ray 13

3.6.2. Blood Tests 13

3.6.3. Assessment of Patient Outcomes 13

3.7. Study Procedures 14

3.7.1. Recruitment of Participants 14

3.7.2. Baseline Visit 14

3.7.3. Randomisation 14

3.7.4. Follow-Up 14

3.7.5. Laboratory Procedures 15

3.7.6. Training of Study Staff 15

3.7.7. Trial Registration 15

3.7.8. Definition of End of Trial 15

3.7.9. Discontinuation/Withdrawal of Participants from Study 15

4. SAFETY 15

4.1. Adverse Event Reporting 16

4.2. Definition of an Adverse Event 16

4.3. Definition of a Serious Adverse Event 16

4.4. Adverse Event Monitoring 18

5. STATISTICS 18

5.1. Sample Size 18

5.2. Statistical Analysis 18

6. DATA MANAGEMENT 18

7. ETHICS 18

7.1. Ethics Committee Approval 18

7.2. Participant Consent 18

7.3. Participant Confidentiality 19

8. PUBLICATION POLICY 19

9. REFERENCES 20

**1. SYNOPSIS**

| **Study Title** | Effect of adjunct high-dose vitamin D on community acquired pneumonia in hospitalised adults – a randomised controlled trial |
| --- | --- |
| **UTN** | U1111-1140-2885 |
| **ANZCTR No.** | ACTRN12613000582752 |
| **Trial Design** | Randomised, double blind, placebo-controlled trial |
| **Trial Participants** | Adults aged ≥ 18 years old, hospitalised with a confirmed radiological diagnosis of community acquired pneumonia |
| **Planned Sample Size** | up to 400 |
| **Follow-up duration** | 6 weeks post-study treatment |
| **Planned Trial Period** | June 2013-December 2014 |
| **Primary Objective** | To determine whether providing a single high-dose bolus of vitamin D as an adjunct treatment in adults hospitalised with community acquired pneumonia improves the resolution of pulmonary inflammatory infiltrate on chest x-ray compared with placebo. |
| **Secondary Objectives** | To determine whether providing a single high-dose bolus of vitamin D as an adjunct treatment in adults hospitalised with community acquired pneumonia improves patient outcomes compared with placebo. |
| **Primary Endpoint** | The resolution of pulmonary inflammatory infiltrate on a follow-up chest radiograph 6 weeks after taking study treatment. |
| **Secondary Endpoints** | Patient Outcome measures: Length of hospital stay; in hospital mortality; intensive care unit admission rates; amount and duration of antimicrobial therapy; rates of relapse and readmission to hospital after discharge; 6 week post-study treatment mortality rates; 12 month mortality rates; resolution of symptoms and return to normal activity based on participant questionnaire at 6 weeks post-study treatment. |
| **Investigational Medicinal Products** | Vitamin D_3_ |
| **Form** | Tablet |
| **Dose** | A single 200,000 IU, dose within 48 hours of admission to hospital with a confirmed radiological diagnosis of community acquired pneumonia. |
| **Route** | Oral |

**2. BACKGROUND**

**2.1. Community Acquired Pneumonia**

**2.1.1. Epidemiology of Community Acquired Pneumonia**

Community acquired pneumonia (CAP) is an infection of the lower respiratory tract that is acquired outside of hospitals or extended-care facilities. Pneumonia is largely caused by bacteria, particularly *Streptococcus pneumoniae,* which is the major cause of severe CAP and death. It globally accounts for about two-thirds of CAP cases where an aetiological diagnosis is made^1^. Other relatively common causes of CAP include bacteria, such as, *Mycoplasma pneumoniae, Haemophilus influenza*, *Legionella* spp., *Chlamydia pneumoniae* and *Staphylococcus aureus* and viruses, such as, influenza A and parainfluenza viruses. However the order of importance of each pathogen depends on the location and population studied. In New Zealand, *S. pneumoniae, M. pneumoniae, Legionella* spp and *H. influenzae* are the most commonly identified pathogens in adult CAP^2,3^.

Pneumonia affects all age groups however it is a significant cause of mortality in children and older people. In New Zealand, pneumonia is three times more prevalent in Māori than non-Māori^4^. Māori men between the ages of 54-65 years have a mortality rate six times higher than non-Māori, while Māori women aged 65 years and older are three times more likely to be hospitalised^5^. Generally, peak incidence occurs in the winter months, with the exception of pneumonia caused by *Legionella* spp, whose incidence in New Zealand peaks over the spring/summer months; a reflection of the fact that one of the most common sources of infection is from compost and potting mixes.

**2.1.2. Clinical and Economic Importance of Community Acquired Pneumonia**

CAP is a major cause of morbidity and mortality in New Zealand and worldwide. In the United States, pneumonia remains the seventh leading cause of death, with 4-5 million cases of CAP estimated to occur annually, and of these, 25% require hospitalisation^6^. In 2001, the annual cost of CAP in the US was estimated at $8.5 billion^6^. In New Zealand, CAP is the most common cause of admission to hospital for adults and has a reported mortality rate of between 6.5-8%^2-4, 7,8^. In 2003, the estimated incidence rate of CAP in adult New Zealanders was 859 episodes per 100,000 people per year and the cost of treatment and in lost productivity as a result of the disease was valued at $63 million annually^9^.

Pneumococcal vaccination is recommended for the elderly and those who are immunocompromised or with chronic disease. The pneumococcal vaccine has been shown to be 60-70% effective in immunocompromised patients^10^. In addition, pneumococcal vaccination has been associated with reduced virus-associated pneumonia in children^11,12^. Despite this, pneumococcal vaccination is uncommon in New Zealand^3,4^. Immunization against influenza can also play an important role in preventing CAP. There is an abundance of evidence supporting the important interaction between respiratory viruses and bacterial pathogens in the pathogenesis of pneumonia^13^. In particular there is a temporal relationship between the incidence of CAP and the circulation of influenza and respiratory syncytial viruses^14^. The adherence of *S. pneumoniae* to human tracheal epithelial cells is higher in the presence of rhinovirus^15^, and influenza virus infection predisposes to adherence, invasion, and induction of disease by *S. pneumoniae*^13^. In a study of adult CAP in Christchurch, the combination of infection with both rhinovirus and *S. pneumoniae* was independently associated with severe disease (OR 10.0, 95%CI 1.3-75.3)^1^.

### 2.2 Overview of Vitamin D

#### 2.2.1. Vitamin D Metabolism

Vitamin D enters our bodies in two forms: either as ergocalciferol (vitamin D_2_) from plant foods, or cholecalciferol (vitamin D_3_) from sun exposure or consumption of animal foods^16^. Vitamin D_3_ is synthesized in the skin by ultraviolet (UV) B radiation from the sun activating its precursor 7-dehydrocholesterol^16^. For most people, vitamin D status is determined by UVB exposure, which varies according to season at higher latitudes (e.g., more than 35° North or 35° South). Regardless of the source, vitamin D then circulates in the blood to the liver where it is converted to its main form, 25-hydroxyvitamin D (25OHD), which has blood levels about 1000 times higher than the most active metabolite, 1,25-dihydroxyvitamin D (1,25-(OH)_2_D).

#### 2.2.2. 25OHD Levels Required for Optimum Health

Vitamin D status is determined by the measurement of serum 25OHD level. Currently, there is no international consensus regarding what concentration of circulating 25OHD constitutes vitamin D deficiency, and has been a matter of debate for over a decade. Some consider that levels > 75 nmol/L define sufficiency, while others consider 50nmol/L a sufficient level. A recent and controversial report from the Institute of Medicine (IOM) concluded for extra-skeletal outcomes, such as, cancer, cardiovascular disease, diabetes and autoimmune disorders, there was insufficient evidence to show that vitamin D impacted on disease outcome and that 25OHD concentrations of 50 nmol/L were sufficient to maintain the skeletal health of at least 97.5% of the North American population^17^. This is in contrast however to numerous epidemiological studies that suggest that serum 25OHD levels should be 75-100 nmol/L for optimum health. Such studies have observed that the risk of a range of diseases or markers – including bone mineral density, hip fracture, muscle strength, periodontal disease, diabetes, hypertension, coronary heart disease, lung function, colon and breast cancer, and all-cause mortality – is lowest at 25OHD levels >75 nmol/L^18-27^.

**2.2.3. Vitamin D Levels and New Zealand**

Even if the 50 nmol/L cut-off is used to define sufficiency, low vitamin D levels are common world-wide^28^. Vitamin D levels in the New Zealand general population are, on average, lower than at similar latitudes elsewhere in the world. Results from the 2008 New Zealand Adult National Nutritional survey show that just over 27.1% of the population (25.2% men and 28.5% women) have insufficient levels (<50 nmol/L) and nearly 5% are frankly deficient (<25 nmol/L). 25OHD levels are lower in Māori (33.7% insufficient, 6% deficient) and Pacific People (46.6% insufficient, 10.5% deficient) than other ethnic groups. Pacific women have the worst vitamin D status with a reported mean 25OHD concentration of 46.0 nmol/L and over half (53.2%) have insufficient levels. For all adults, mean 25OHD levels are lower in the Southern region compared with Northern or Central parts of New Zealand throughout the year. This is most pronounced over the late winter and early spring months (August-October), where mean levels are 45.5 nmol/L for the Southern region compared with 56.1 nmol/L and 50.5 nmol/L in Northern and Central regions respectively^29^. A Christchurch study demonstrated that most, if not all, of 201 apparently healthy adults randomly selected from the electoral roll did not have adequate circulating 25OHD at some time in the year. Even in February, when UVB exposure is high, only 12% had plasma levels >80 nmol/L^30^. Similarly, a study of over 400 adult CAP patients admitted to either Christchurch (n=300) or Waikato Hospitals (n=123) were found to have median 25OHD levels of 30 nmol/L (28 nmol/L for Christchurch patients and 34 nmol/L for Waikato patients) and 26% exhibited severe deficiency with levels ≤ 20 nmol/L (unpublished).

The most likely explanation for the lower than expected vitamin D status in New Zealand is the Cancer Society’s longstanding and widely-accepted advice to avoid sun exposure to prevent skin cancer. With one of the highest malignant melanoma rates in the world^31^, New Zealanders are very aware of sunlight exposure. The food supply in New Zealand is also minimally fortified with vitamin D, compared to other countries such as the USA^32^. Vitamin D supplements in New Zealand are mainly available in small doses (mostly 100 IU multivitamin tablets) which only increase serum 25OHD levels by 1-2 nmol/L^33^.Thus, current over-the-counter oral sources of vitamin D available to the general population in New Zealand are only able to increase serum 25OHD by very small amounts.

**2.2.4. Vitamin D Supplementation, Dosing Regimes and Absorption**

The purpose of the current IOM recommendations for the daily intake of vitamin D are to maintain skeletal health by preventing rickets/osteomalacia in the general, healthy population and aim to keep circulating 25OHD levels at or above 50 nmol/L. Recommended doses are 600 IU per day for those aged between 1 and 70 years of age and 800 IU for those aged over 70 years^17^. However, much higher doses are required to achieve the vitamin D levels considered, at least by some, to be optimal (i.e. >75-100 nmol/L). For example, to raise serum 25OHD from 50 to 80 nmol/L requires an additional 1700 IU of vitamin D_3_ per day^34^, while a dose of 4000 IU vitamin D_3_ per day is required to raise serum 25OHD levels of some individuals to 100 nmol/L^35^. In addition, higher doses are likely to be required for those people with chronic illnesses; kidney disease, liver disease, Crohn’s disease or other diseases associated with malabsorption of nutrients. In these instances, uncertainty exists on the dosage and regime that should be used.

Since the 1930’s it has been assumed that vitamin D_2_ and vitamin D_3_ have the same efficacy in raising 25OHD concentrations, but recent data has questioned the validity of this assumption. Of the limited evidence currently available, it appears that vitamin D_2_ is less efficacious than vitamin D_3_ when given in large monthly or single bolus doses^36-38^, yet when either form of the vitamin is given in smaller daily doses, circulating 25OHD levels increase to the same degree^36^. Studies have shown that following a single large bolus dose (50,000, 100,000 or 300,000 IU) of either vitamin D_2_ or D_3_ there is a similar rapid and significant rise in 25OHD concentrations over the first 3 days, indicating equivalent absorption. After which, 25OHD levels continue to rise following vitamin D_3_ supplementation, peaking between 7 and 14 days before declining, with it taking >60 days for 25OHD levels to return to pre-supplementation levels^37,39^. However, following vitamin D_2_ supplementation, 25OHD concentrations fall rapidly after 3 days and are not significantly different from pre-supplementation levels at 14 days^36-38^. The same pattern is observed when vitamin D_2_ or D_3_ is provided intramuscularly, with a single large dose of vitamin D_3_ being almost twice as potent as D_2,_ however, the rise in 25OHD concentrations is far greater for both forms of the vitamin when given orally^38^.

### 2.3. Vitamin D and Respiratory Infection

#### 2.3.1. Vitamin D Activates Cathelicidin Production and Suppresses Inflammation in Respiratory Infections

Recently, vitamin D has been shown to have an important role in the innate immune system, which prevents infection without the need for immunological memory from previous exposure to the pathogen^40^. Innate immunity includes the production of antimicrobial peptides that are capable of killing viruses, bacteria and other organisms^41,42^. These peptides are produced on epithelial surfaces and within circulating white blood cells. Examples include human β-defensins 2 and 3 and cathelicidin (also known as hCAP-18 and LL-37)^42^. The peptides are produced through the effect of Toll like receptors, on the surface of macrophages and monocytes, causing cell activation when they recognize molecules derived from pathogens^42,43^. This activation results in expression of the genes that code for the vitamin D receptor, and for the 1α-hydroxylase enzyme that converts the pre-hormone, 25OHD, to the biologically active 1,25 dihydroxyvitamin D (1,25(OH)_2_D); which in turn activates the gene that produces cathelicidin^43^. An increase in the concentration of cathelicidin in phagocytic vacuoles enhances the cells ability to kill microorganisms^42^. LL-37, the only human cathelicidin, has been identified in the following tissues: white cells, breast milk, skin, lung, saliva, and colon; and is active against a wide range of microbes, including bacteria (both gram positive and gram negative), fungi and viruses^44^.

Vitamin D also has an essential role in the adaptive immune system, which is important for immunological memory and the production of antigen-specific antibodies. 1,25(OH)_2_D3 is important in the maturation of dendritic cells, which are one of the major antigen presenting cells that capture, process and present antigens to T cells that in turn, are responsible for coordinating and enhancing the adaptive immune response to a particular pathogen. In addition, 1,25(OH)_2_D modulates the cytokine profile of antigen presenting cells by suppressing pro-inflammatory cytokines and increasing the expression of cytokines, such as, IL-10, which have broad spectrum anti-inflammatory activities^45^. This reduces inflammation and unwanted tissue damage that can be caused by an over-exuberant or unregulated immune response to a pathogen, rather than damage caused by the pathogen itself.

**2.3.2. Vitamin D and Tuberculosis**

The new evidence linking vitamin D and cathelicidin^43^ provides a possible explanation for the link between sun exposure, vitamin D and tuberculosis (TB)^46^. There is increasing evidence that low body vitamin D levels may increase the risk of developing TB. For example, a hospital-based case-control study in London found that vitamin D deficiency was associated with an odds ratio of 2.9 (95%CI 1.3-6.5) for having active TB^47^. A case series of London TB patients showed that 56% had undetectable plasma 25OHD levels (<7 nmol/L)^48^. A case-control study from West Africa observed lower mean serum 25OHD levels in cases (78 nmol/L) compared with controls (85 nmol/L; p<0.001)^49^. Susceptibility to TB has been linked to vitamin D-receptor polymorphisms, with the presence of the *Fok*I F allele protecting against TB infection, and the *Taq*I t allele protecting against active disease but not infection^50^.

#### 2.3.3. Epidemiological Studies of Rickets, Vitamin D and Respiratory Infection

The association between rickets and infection has been known since the 1960s^51^. Since then, numerous studies have reported that children with rickets commonly present to hospital with respiratory infections^52-60^. One of these studies observed lower serum 25OHD levels in cases of acute severe lower respiratory infection requiring admission to hospital compared with controls: 23 *vs* 38 nmol/L (p<0.0001)^60^.

Exposure to sunshine and UVB, which is the primary source of vitamin D in humans,^61^ is also associated with respiratory infection markers. Sub-erythemal courses of UV radiation, administered twice a year for three years to Russian teenage athletes, resulted in fewer respiratory viral infections, fewer days of absences and shorter duration of illness, compared with non-irradiated athletes^62^. The irradiated subjects also had significant increases in salivary IgA, IgG and IgM compared with controls. A Dutch study found that children with low sun exposure were more likely to have a cough and a runny nose, compared to children with most sun-exposure^63^. The temporal association between seasonal changes in vitamin D levels and winter respiratory virus activity has led to the proposal that low vitamin D levels may have a causal role in the onset of influenza epidemics^64,65^.

Several observational studies have reported on vitamin D status and respiratory infection. A Turkish case-control study found that serum 25OHD levels were lower in neonatal cases of acute lower respiratory infection (23 nmol/L) than age-matched controls (41 nmol/L)^66^. A Finnish cohort study found that young male soldiers with serum 25OHD levels <40 nmol/L at baseline had a 63% increased risk of absence from duty over the following 6 months as a result of a respiratory infection compared with soldiers with levels >40 nmol/L (P=0.004)^67^. A Canadian case-control study of children aged 1-25 months found no difference in mean serum 25OHD levels between cases of acute lower respiratory tract infection (77 nmol/L) and hospital controls (77 nmol/L)^68^. However this finding is probably a result of virtually all of the infants having high vitamin D diets through fortified formula or supplementation. A secondary analysis of the US NHANES III survey showed that, after adjusting for demographic and clinical characteristics, lower 25OHD levels were independently associated with self-reported upper respiratory tract infection (URI; cold and flu-like illnesses) in the past few days (compared to ≥75 nmol/L group: OR 1.36 [95%CI, 1.01-1.84] for <25 nmol/L and OR 1.24 [95%CI, 1.07-1.43] for 25-74 nmol/L groups)^69^. In a New Zealand cohort of 112 patients admitted to hospital with CAP, median 25OHD levels were 54 nmol/L, 15 % had serum levels <30 nmol/L and low levels were significantly associated with a higher 30-day mortality (OR 12.7; [95%CI 2.2-73.3])^70^. Similarly, in a cohort consisting of over 400 adult CAP patients admitted to either Christchurch or Waikato hospitals, 67% of those with 25OHD levels > 50 nmol/L had complete resolution of pulmonary infiltrate as assessed by chest x-ray at 6 weeks after hospital discharge, while only 53% of patients with levels <25 nmol/L and 56% of patients with levels between 25-50 nmol/L had complete resolution. In addition, a higher mortality rate (11%) was observed for those patients that had 25OHD levels < 25 nmol/L and fewer reported being back to normal activity (50%) compared to those that had levels > 50 nmol/L (4% mortality and 61% reported return to normal activity)(unpublished).

Observational studies of vitamin D and respiratory infection are likely to be affected by biases in measurement of both the relevant vitamin D level and the outcome, as well as potential confounding by exposure to sunlight (e.g. ‘sickly’ children might be less likely to spend time uncovered outside). Strong evidence of whether there is an effect of vitamin D on the risk of infection can be generated only by randomised controlled trials.

#### 2.3.4. Trials of Vitamin D Supplementation and Respiratory Infection

Two randomised controlled trials (RCTs) of vitamin D supplementation have indirectly examined its impact on URI’s. A study to prevent bone-loss in post-menopausal African-American women found that 8% of women on 800 to 2000 IU per day reported having cold or influenza symptoms over the 3 years follow-up compared with 25% of women on placebo (p<0.002)^71^. The prevalence of URI symptoms in this study is undoubtedly underestimated because of the insensitive and imprecise manner in which these data were collected, although the double-blinded, randomised study design should have minimised reporting bias. In a sub-study of an RCT to prevent fractures with vitamin D supplementation, 3444 participants (mean age 77 years) were asked in winter if they had suffered an infection or received antibiotics during the previous week^72^. For intention-to-treat comparisons, there was a non-significant 10% reduction in the odds of reporting infection (p=0.23) and 16% reduction in the odds of reporting antibiotic use (p=0.18). Slightly stronger effects were observed for on-treatment per-protocol comparisons: 20% reduction in reporting infection (p=0.06) and 26% reduction in reporting antibiotics (p=0.10). Limitations of this study include the short outcome period of only one week, which reduced power, and its low dose of vitamin D (800 IU/day) which increased 25OHD levels from 38 nmol/L to only 62 nmol/L, well below the 80 to 100 nmol/L range now considered by some to be associated with optimum adult health outcomes^18,19,25,26^.

Four RCT’s have directly examined the effect of vitamin D supplementation on the risk of respiratory tract infections. One study with 162 adults showed no benefit of vitamin D supplementation in decreasing the incidence or severity of URIs during winter^73^. However, this study was of short duration (12 weeks), underpowered and used a relatively low dose of vitamin D (2,000 IU daily) without a loading dose. Despite these short-comings, a more recent study with 322 adults also showed no benefit of vitamin D supplementation on the incidence or severity of URI’s. In this instance, the study was adequately powered, was over an 18-month period including two winters, used a high dose of vitamin D (100,000 IU’s monthly) and participants were given a loading dose of 200,000 IU/month for the first two months of the study^74^. However, this study population did not have a high prevalence of deficiency, where even over the winter months the mean serum 25OHD concentration was about 50 nmol/L in the placebo group. In contrast to these findings another recent RCT with 744 children showed that in a subgroup analysis of one-third of the total cohort (n = 247) a daily dose of milk fortified with 300 IU vitamin D reduced the risk of respiratory infections by half compared to those that received unfortified milk (placebo group)^75^. In this study population however, there was a high prevalence of vitamin D deficiency, with median baseline levels of 17.5 nmol/L. Similarly, when the analysis included the whole cohort the effect was no longer significant. One further RCT with over 3000 infants (1-11 months of age) has directly examined the effect of vitamin D supplementation (100,000 IU quarterly) on lower respiratory tract infections and showed no benefit of supplementation for reducing the incidence of pneumonia^76^. Further research is still required to determine if there is benefit in other populations.

**2.3.5. Vitamin D as an Adjunct Therapy**

Recently there has been a limited number of RCT’s reporting the use of vitamin D_3_ supplementation as an adjunct to the conventional treatment for a various diseases including TB, multiple sclerosis (MS), cystic fibrosis (CF) and pneumonia. In one study with 146 newly diagnosed TB patients, 4 fortnightly doses of 100,000 IU vitamin D_3_ in addition to conventional TB treatment did not significantly improve the median time to sputum culture conversion^77^. However, a subgroup per-protocol analysis of the 95 patients that completed the trial on study treatment showed that vitamin D supplementation not only significantly accelerated sputum culture conversion but also enhanced the resolution of the inflammatory response by numerous immunomodulatory effects including improving the lymphocyte count and suppressing pro-inflammatory cytokines^78^. Similarly, in a further study, supplementation with high doses of vitamin D (two doses of 600,000 IU’s) accelerated clinical and radiographic improvement in all TB patients and for the patients that were vitamin D deficient prior to treatment, host immune system function also improved^79^. In contrast, another trial found that in patients with active TB, vitamin D supplementation of 100,000 IU at enrolment, 5 and 8 months of study did not reduce the clinical severity score, time to sputum culture conversion or improve 12 month mortality rates^80^. However a significant limitation of this study is that the dose of vitamin D may have been insufficient to see an effect.

Two studies have examined the effect of vitamin D adjunct therapy in MS. Vitamin D supplementation of 20,000 IU per week for 12 months was shown to reduce the number of T1 enhancing lesions, the T2 burden of disease and also disability accumulation compared to those on placebo, although the latter two effects did not reach statistical significance (p = 0.105 and 0.071, respectively)^81^. This is likely to be because of the small study size of only 66 patients with a resulting lack of power to detect significant differences between the treatment arms for most of the primary and secondary end-points. In contrast, a pilot study with 50 MS patients showed that low dose vitamin D (10 IU per day for two weeks, followed by 20 IU per day for 12 months) had no effect on the expanded disability score or relapse rate^82^. The major limitation of this study however is the low dose of vitamin D.

In a pilot study with 30 CF patients who were admitted to hospital with pulmonary exacerbation, a single bolus oral dose of 250,000 IU vitamin D significantly increased the unadjusted 1 year survival and number of hospital free days and there was also a trend towards increased intra-venous antibiotic free days^83^.

Two RCT’s in children have examined the effect of vitamin D supplementation as an adjunct therapy for pneumonia. In conjunction with conventional antibiotic treatment in children under 5 years of age hospitalised with severe pneumonia, one study with 200 patients, gave either 1000 IU vitamin D to those aged < 1 year old, 2000 IU vitamin D to those aged >1 year old or placebo daily for 5 days. No beneficial effect of short-term vitamin D supplementation was found on the resolution of severe pneumonia. Similarly, the duration of hospitalisation, time to resolution of tachypnea, chest restrictions and an inability to feed was similar between the supplemented and placebo groups^84^. The limitations of this study however, may be that the dose of vitamin D was insufficient. Another study with 453 infants aged 1-36 months with confirmed pneumonia were given either a single bolus dose of 100,000 IU vitamin D_3_ or placebo in addition to antibiotics. There was no significant difference in the mean number of days to recovery between the two groups, however, the risk of a repeat episode of pneumonia within 90 days of supplementation was significantly reduced in those given vitamin D (RR 0.73 [CI 0.64-0.94], P=0.01)^85^.

Although some of these trials indicate that vitamin D supplementation may be beneficial, several studies are underpowered or use low dose vitamin D. Further trials are needed to address these shortcomings to be able to appropriately test the efficacy of vitamin D supplementation as an adjunct therapy.

**2.4. Genetics Sub-study**

Genes significantly influence vitamin D status (29-43%) and polymorphisms in vitamin D pathway genes (especially the vitamin D receptor (VDR) gene) have been associated with a variety of diseases^86^, including respiratory tract infections^87^. Although most studies to date have examined only a few *VDR* polymorphisms, more are beginning to comprehensively investigate polymorphisms in the *VDR* as well as in other vitamin D pathway genes, such as the vitamin D–binding protein gene (*GC*), 7-dehydrocholesterol reductase (*DHCR7*), *CYP27B1* and *CYP24A1*. These genes encode enzymes that, respectively, synthesize and degrade 1α,25-(OH)_2_-vitamin D and are the genes that have been most strongly linked to circulating 25OHD levels^88,89^. There is limited evidence suggesting that individuals carrying risk alleles for lower 25OHD concentrations have an enhanced response to supplementation^90^, but this area is largely unexplored.

Although genetic analysis is outside the scope of this study, we will collect an additional tube of blood at baseline from consenting participants, which will be stored for future genetic analysis to determine the impact of genetic factors on the response to supplementation.

**3. RESEARCH DESIGN AND METHODS**

**3.1. Hypothesis**

The main hypothesis of this project is that providing vitamin D as an adjunct to standard antibiotic therapy will accelerate the resolution of pulmonary inflammatory infiltrate in CAP.

The specific objectives are to determine whether providing a single, oral, high-dose bolus of vitamin D in adults hospitalised with CAP will:

1. Improve the resolution of pulmonary inflammatory infiltrate compared with placebo.

2. Improve patient outcomes compared with placebo.

**3.2. Study Overview**

The study is a randomised, double blind, placebo-controlled trial in adults admitted to hospital with a confirmed radiological diagnosis of CAP. Participants will be randomised to receive a single oral dose of 200,000 IU vitamin D_3_ or matching placebo within 48 hours of admission to hospital. The primary outcome measure will be the resolution of pulmonary inflammatory infiltrate on chest x-ray at 6 weeks (± 1 week) after receiving study treatment. Secondary outcomes will include measures of patient outcomes including; length of hospital stay, in hospital mortality, intensive care admission rates, rates of relapse and readmission to hospital after discharge, 6 week post-study treatment mortality rates, 12-month mortality rates and the resolution of symptoms and return to normal activity at 6 weeks post-study treatment as assessed from hospital records and a structured telephone interview.

**3.3. Participants**

Participants will be recruited from CAP patients admitted to Christchurch Hospital and Princess Margret Hospital sites. On average, 400 to 500 people are admitted to these hospitals annually with a diagnosis of CAP, with the peak incidence occurring over the winter months (June to August).

Inclusion criteria:

1. A confirmed radiological diagnosis of pneumonia, defined as:

‘New inflammatory infiltrate on a chest radiograph where patients have an acute illness with clinical features of pneumonia and radiographic pulmonary shadowing that is at least segmental or present in one lobe and is neither pre-existing or because of some other known cause.’

1. <48 hours since admission to hospital
2. Age ≥ 18 years
3. Ability to give informed consent
4. Ability to orally consume the study medication
5. Ability to attend a follow-up appointment at 6 weeks post-study treatment for a chest x-ray

Exclusion criteria:

1. Pneumonia is not the principal reason for admission
2. Pneumonia associated with bronchial obstruction, bronchiectasis, or known tuberculosis
3. Use of vitamin D_3_ supplements other than as part of a multivitamin preparation where the daily intake would not exceed 400 IU.
4. High plasma corrected calcium > 2.6 mmol/L (corrected for plasma albumin concentration)
5. Use of immunosuppressants (e.g. doses of prednisone >10mg, methotrexate, azathioprine and cyclosporin)
6. Any prior history of hypercalcaemia, nephrolithiasis or sarcoidosis
7. Current kidney disorders requiring dialysis treatment or polycystic kidney disease
8. Cirrhosis of the liver
9. Women who are pregnant or are breastfeeding*
10. Already enrolled or planning to enrol in a research study that would conflict with full participation in the study or confound the observation or interpretation of the study findings (e.g. where vitamin D levels are tested and results are known by the participant; where the participant is required to take conflicting medications)

* At the 6 week follow-up women of reproductive age that think/know they are pregnant will also be excluded from taking further part in the study.

**3.4. Intervention**

Participants randomised to the active treatment will receive a single oral dose of 200,000 IU vitamin D_3_ < 48 hours of admission to hospital with a confirmed radiological diagnosis of CAP. Those randomised to placebo will receive matching inactive tablets that will be administered in an identical regimen. The vitamin D_3_ and matching placebo tablets will be sourced from Tishcon Corp (an accredited pharmaceutical company in New Jersey, USA). All treatments will be directly administered to participants by research staff upon enrolment into the study.

The dose of 200,000 IU vitamin D was chosen to rapidly increase serum 25OHD levels to approximately 80-100 nmol/L and is based on data from two studies that have assessed the pharmacokinetics and safety of high dose vitamin D_3_. One study in healthy adults showed that a single oral dose of 100,000 IU vitamin D_3_ increased plasma 25OHD levels from around 65 nmol/L to ≥ 80 nmol/L within 24 hours and reached a maximum of approximately 100 nmol/L at 7 days following consumption^39^. A second study in elderly women showed that a single oral dose of 300,000 IU sharply increased 25OHD levels from approximately 30 nmol/L to 125 nmol/L after 3 days and levels reached a maximum of approximately 150 nmol/L within 30 days of consumption before decreasing^38^. There were no safety issues or incidences of hypercalcaemia reported for either study. Similarly, two further studies that used single large bolus doses of 500,000 IU vitamin D showed that 25OHD levels were between 150-210 nmol/L one month following supplementation and neither study reported any incidences of hypercalcaemia^91,92^. Thus, large single doses of vitamin D_3_ up to 500,000 IU have been shown to be safe and effective for rapidly raising 25OHD levels above 80 nmol/L.

**3.5. Study Period**

The study will be conducted from June 2013 to December 2014 or until the sample size has been reached. Recruitment will be conducted as potential participants are admitted to hospital, identified and enrolled. Participants will be enrolled and receive their study treatment < 48 hours of admission to hospital. All participants will be observed from the time of the study treatment being given until 6 weeks (± 1 week) after receiving study treatment. This observation period was chosen as generally, 6 weeks is the standard clinical time point utilised for patients that have had pneumonia to assess its resolution by chest x-ray.

**3.6. Data Collection**

**3.6.1. Chest X-Ray**

The main outcome measure will be the resolution of pulmonary inflammatory infiltrate. Participants will be asked to undergo a follow-up chest x-ray, 6 weeks (± 1 week) after receiving study treatment.

**3.6.2. Blood Tests**

Blood samples will be collected at recruitment and at 6 weeks (± 1 week) after receiving study treatment and will be stored. Vitamin D (25OHD) levels will be measured on baseline blood samples at the end of the 6 week follow-up period to assess the proportion of people recruited into the study with deficient levels (<25 nmol/L). These baseline measures will be fed-back to the participant’s GP who will be responsible for the on-going care of the participant. The final blood sample collected at the end of the 6 week post- study treatment follow-up period will be stored and 25OHD levels measured after all participants have completed the 6 week follow-up period so as not to compromise the blinding of participants or research personnel. Plasma calcium and albumin levels will be measured in real-time on all samples collected and the corrected plasma calcium (i.e. corrected for plasma albumin concentration, as is standard laboratory practice) will be reported in order to check for hypercalcaemia (an elevated plasma corrected calcium is > 2.6 mmol/L). Only the corrected plasma calcium levels will be made available to investigators before the end of the 6 week follow-up period.

**3.6.3. Assessment of Patient Outcomes**

Clinical data including basic demographics, routine haematological and biochemical information, length of hospital stay, in-hospital mortality, intensive care admission rates, amount and duration of antimicrobial therapy and rates of relapse and readmission to hospital and mortality rates at 6 weeks post-study treatment will be obtained from patient hospital records. For all participants, a CURB65 pneumonia severity score will be calculated from the clinical data collected. Research staff will also conduct a telephone interview with study participants at 6 weeks post-study treatment to complete a questionnaire regarding the resolution of symptoms and if the participants have returned to normal activity. Twelve-month mortality rates will be obtained from national databases.

**3.7. Study Procedures**

**3.7.1. Recruitment of Participants**

All patients with a clincial diagnosis of pneumonia on admission to Christchurch Hospital are notified to a centralised patient registry which will be reviewed twice daily by members of the research team to identify potential participants. For these patients, computer records in the Depatment of Radiology will be selected electronically using an algorithm utilising key words such as pneumonia, consolidation and inflammatory infiltrate and the radiographs will be reviewed by a consultant radiologist to confirm the radiological findings. New admissions for CAP will be identified by study personnel on a daily basis on weekdays (Monday-Friday). Once identified, patients will be approached and asked to participate in the study within 48 hours of admission.

The study population will be comprised of at least the first 360 CAP patients that provide informed consent to participate (n= 180 per arm). However, to increase the possibility of seeing an effect, we aim to have ≈ 30% (n = 120) of the study cohort to be participants who are vitamin D deficient at baseline (25OHD levels <25 nmol/L). Thus, recruitment may need to continue to get the required numbers of deficient patients or until a maximum of 400 participants have been enrolled.

**3.7.2. Baseline Visit**

The study will be explained to potential participants by trained research staff. After informed consent has been obtained and eligibility has been assessed, all participants will be provisionally enrolled in the study until the result of the corrected plasma calcium level is known. Only those patients that do not have hypercalcaemia (corrected plasma calcium > 2.6 mmol/L) will be fully enrolled and randomised to receive vitamin D_3_ or placebo. Clinical data (including basic demographics, biochemical measures and CURB65 severity score) will be obtained from the patients’ hospital records and confirmed as needed by patient enquiry. A blood sample for 25OHD measurement will also be obtained at baseline.

**3.7.3. Randomisation**

The sequence numbers 1 to 500 will be allocated, with equal probability, to Vitamin D treatment and placebo treatment by a block random number generator (R Vienna Austria). Labelled bottles with the appropriate contents will be arranged in numerical order and the one with the lowest number among those unallocated will be used for the next recruited participant. Only the study statistician will have access to the randomisation codes and he will also be responsible for organising the preparation of the study treatment in conjunction with the Christchurch Hospital pharmacy dispensary. No research personnel that will have contact with the study participants will be involved in the preparation of the treatment, thus ensuring that those running the trial and the study participants remain blind to allocation.

**3.7.4. Follow-up**

At 6 weeks (± 1 week) post-study treatment all participants will be required to undergo a chest x-ray and provide a further blood sample for plasma calcium and serum 25OHD measures. Any women of reproductive age who thinks/knows they have become pregnant within the 6-week follow-up period will be excluded from further participation in the study and will not have a chest x-ray at 6 weeks. All participants will also be contacted by research staff at 6 weeks post-study treatment to answer a questionnaire regarding the resolution of symptoms and the return to normal activity.

Clinical data including length of hospital stay, in-hospital mortality rates, mortality rates at 6 weeks post-study treatment, intensive care admission rates, amount and duration of antimicrobial therapy and rates of relapse and readmission to hospital will be obtained from patient hospital records at 6 weeks post-study treatment. Twelve-month mortality rates will be obtained from national databases. This data will be analysed and reported subsequently to that of the 6 week follow-up data so the analysis and publication of the primary study findings is not delayed.

**3.7.5. Laboratory Procedures**

All laboratory procedures will be performed at Canterbury Health Laboratories under the supervision of Prof David Murdoch.

**3.7.6. Training of Study Staff**

Training and education sessions for study personnel will be conducted in the month prior to the recruitment start date. The training will cover all study procedures, including recruitment and consenting as well as interviewing methods.

**3.7.7. Trial Registration**

The trial has been registered with the Australian and New Zealand Clinical Trials Registry; ACTRN12613000582752; http://www.ANZCTR.org.au

**3.7.8. Definition of the End of the Trial**

The end of the trial is the 6 week (± 1 week) post-study treatment follow-up chest x-ray and questionnaire completion regarding the resolution of symptoms and return to normal activity of the last participant.

**3.7.9. Discontinuation/Withdrawal of Participants from the Study**

Each participant has the right to withdraw from the study at any time. In addition, the investigator may discontinue a participant from the study at any time if the investigator considers it necessary for any reason including:

- Ineligibility (either arising during the study or retrospective having been overlooked at screening)
- Significant protocol deviation
- Significant non-compliance with study requirements
- An adverse event which results in inability to continue to comply with study procedures
- Corrected plasma calcium levels >2.6 mmol/L (see safety below)
- Disease progression which results in inability to continue to comply with study procedures
- Consent withdrawn
- Lost to follow up

The reason for withdrawal will be recorded. If the participant is withdrawn due to an adverse event, the investigator will arrange for follow-up visits or telephone calls until the adverse event has resolved or stabilised.

**4. SAFETY**

Vitamin D intoxication, which is potentially fatal, is very rare, but can be caused by the ingestion of very high doses. The reported instances have been usually associated with prolonged intakes of more than 40,000 IU per day^93^. Although current data support the viewpoint that serum 25OHD levels must rise above 750 nmol/L to produce vitamin D toxicity, an upper limit of 250 nmol/L is used to ensure a wide safety margin^94^. Prolonged, continued oral intake in excess of 10,000 IU per day is required to produce a serum 25OHD of ≥250 nmol/L. A single high dose bolus of either 100,000, 300,000 or 500,000 IU vitamin D_3_ has been shown to be safe and effective in raising 25OHD levels in both healthy adults and elderly patients, with no reported incidences of intoxication^38,39,91^.

**4.1. Adverse Event Reporting**

Safety of exposure to vitamin D will be evaluated by the incidence of adverse events (AEs) and serious adverse events (SAEs), which will be reported in tabular form giving absolute numbers and percentages. Clinical oversight will be provided by investigators David Murdoch and Stephen Chambers. They will be responsible for assessing causality and whether the AE meets the criteria for an SAE. Each AE and SAE will be classified using the Common Terminology Criteria for Adverse Events (version 4.02; US department of Health and Human Sciences). All observed or volunteered AEs regardless of treatment group or suspected causal relationship to the investigational product will be reported as described in the following sections.

For all AEs, the investigators must pursue and obtain information adequate both to determine the outcome of the AE and to assess whether it meets the criteria for classification as a SAE requiring immediate notification to the designated representative. For all AEs, sufficient information should be obtained by the investigator to determine the causality of the AE. For AEs with a causal relationship to the investigational product, follow-up by the investigator is required until the event or its sequelae resolve or stabilize at a level acceptable to the investigator.

**4.2. Definition of an Adverse Event**

It is the responsibility of the investigators to ensure that all AEs, and other clinically significant findings that occur, are documented and accurately reported. It is important that all site staff understand the requirements and responsibilities related to safety reporting outlined below.

An AE is any untoward medical occurrence in a clinical investigation subject administered a product or medical device; the event need not necessarily have a causal relationship with the treatment or usage. Examples of AEs include but are not limited to:

- - Abnormal test findings
  - Clinically significant symptoms and signs
  - Changes in physical examination findings
  - Hypersensitivity
  - Progression/worsening of underlying disease
  - Additionally, they may include the signs or symptoms resulting from:
  - Drug overdose
  - Drug withdrawal
  - Drug abuse
  - Drug misuse
  - Drug interactions
  - Drug dependency

**4.3. Definition of a Serious Adverse Event**

A SAE or serious adverse drug reaction is any untoward medical occurrence at any dose that:

- - Results in death
  - Is life-threatening
  - Requires inpatient hospitalisation
  - Results in persistent or significant disability/incapacity
  - Results in congenital anomaly/birth defect

Medical and scientific judgment should be exercised in determining whether an event is an important medical event. An important medical event may not be immediately life threatening and/or result in death or hospitalisation. However, if it is determined that the event may jeopardise the subject and may require intervention to prevent one of the other AE outcomes, the important medical event should be reported as serious. Examples of such events include allergic broncho-spasm requiring intensive treatment in an emergency room or at home, blood dyscrasias or convulsions that do not result in hospitalization, or development of drug dependency or drug abuse.

Life-threatening refers to immediate risk of death as the event occurred per the reporter. A life-threatening experience does not include an experience, had it occurred in a more severe form, might have caused death, but as it actually occurred, did not create an immediate risk of death. For example, hepatitis that resolved without evidence of hepatic failure would not be considered life-threatening, even though hepatitis of a more severe nature can be fatal. Similarly, an allergic reaction resulting in angioedema of the face would not be life-threatening, even though angioedema of the larynx, allergic bronchospasm, or anaphylaxis can be fatal.

Hospitalisation is official admission to a hospital. Hospitalisation or prolongation of a hospitalisation constitutes criteria for an AE to be serious; however, it is not in itself considered a SAE. In the absence of an AE, a hospitalisation or prolongation of a hospitalisation should not be reported as an SAE. This is the case in the following situations:

- The hospitalisation or prolongation of hospitalisation is needed for a procedure required by the protocol.
- The hospitalisation or prolongation of hospitalisation is part of a routine procedure followed by the centre (eg, stent removal after surgery). This should be recorded in the medical record.

In addition, hospitalisation, for a pre-existing condition that has not worsened, does not constitute an SAE.

Disability is defined as a substantial disruption in a person’s ability to conduct normal life functions.

The relationship of the AE to Study Medication will be specified as follows:

1. Not related. In the investigators opinion, there is not a causal relationship between study medication and the AE.
2. Unlikely. Study medication is not likely to have any reasonable association with the AE.
3. Possibly. The AE could have been produced by the participant’s clinical state or study medication.
4. Probably. The AE follows a reasonable temporal sequence from the time of study medication administration and cannot be reasonably explained by the known characteristics of the clinical state.
5. Definitely. The AE follows a reasonable temporal sequence from the time of study medication administration.

AEs will be collected from the time of study drug administration through the follow-up period at 6 weeks post-study treatment. The investigators should attempt, if possible, to establish a diagnosis based on the presenting signs and symptoms. In such cases the diagnosis should be documented as the AE and not the individual sign/symptom. If a clear diagnosis cannot be established, each sign and symptom must be recorded individually.

The investigators should attempt to follow all unresolved AEs and/or SAEs observed during the study until they are resolved or judged medically stable, or are otherwise medically explained. If there is any doubt about whether the information constitutes a SAE, the information is treated as a SAE.

Certain information, although not considered an SAE, must also be recorded, reported, and followed up as indicated for an SAE. This includes:

- Overdose of an investigational product as specified in this protocol with or without an AE.
- Inadvertent or accidental exposure with or without an AE.

**4.4. Adverse Event Monitoring**

An independent study data monitoring committee consisting of between 3-4 Canterbury District Health Board specialist physicians will serve as the data and safety monitoring board for the study. A majority of the committee will have had prior experience in data/safety monitoring. Dr John Pearson, the study statistician will liaise with the committee and produce both open and closed reports for the committee meetings that will occur at least every six months. The committee will be notified within 24h of the investigators awareness of any SAE, and will be kept informed of the situation and its development on a continuing basis.

**5. STATISTICS**

**5.1. Sample Size**

The primary outcome measure is to compare the resolution of inflammatory infiltrate on a follow-up chest x-ray at 6 weeks post-study treatment between the vitamin D and placebo arms. These sample size calculations are based on an uncorrected chi square test. To detect an increase from 50% to 70% for clear chest x-rays and assuming an 80% retention rate, 180 participants per arm would be required for a power of 0.8. In addition, we aim to have a high proportion of deficient participants within the study cohort to increase the possibility of seeing an effect. We have calculated we will need to recruit ≈120 participants with deficient vitamin D levels at baseline (i.e. < 25 nmol/L). Thus, based on our previous findings and the data from the National Nutritional Survey^29^ up to 400 CAP patients may need to be recruited to get a sufficient number of deficient participants.

**5.2. Statistical Analysis**

All randomised participants will be included in the primary analysis. Data will be analysed on an intention to treat basis with dropout events reported on and examined for differential compliance between treatment and placebo arms. Primary analysis will be logistic regression of clear chest X-ray on treatment arm with covariates for age (grouped) and gender and if appropriate severity and comorbidities. Additional analyses will include covariates for initial 25OHD level and 25OHD level during the study and stratified analyses by 25OHD level at baseline; deficient (<25nm/l) and insufficient (<50nm/L). All data will be analysed in R (Vienna, Austria) or SAS (NC,USA).

**6. DATA MANAGEMENT**

All data will be double-entered into an electronic database by trained research staff. Macros and interim tabulations will be used to check data ranges and consistency.

**7. ETHICS**

**7.1. Ethics Committee Approval**

The protocol, informed consent form, participant information sheet, questionnaires and proposed advertising material has been approved by the Southern Health and Disability Ethics Committee-13/STH/41. The Investigator will submit and, where necessary, obtain approval from the committee for all substantial amendments to the original approved documents.

**7.2. Participant Consent**

Potential participants asked to participate in the study are entitled to choose whether or not to take part. Their decision is voluntary and they should be competent to understand what is involved. Consent forms are designed to assure the protection of participant rights. Written informed consent will be obtained from each participant before enrolment in the study.

Participants will receive adequate verbal and written information in English. All of our forms and questionnaires are written in English and we are not confident we can get adequate interpreters for other languages in accordance with good clinical practice, therefore it will be a requirement that all participants are able to comprehend and communicate in English. This language requirement should not unduly disadvantage any ethnic group from participating in the study. The verbal explanation will cover all the elements specified in the written information provided for patients. The investigator or co-worker will inform the patient of the aims, methods, anticipated benefits and potential hazards of the study including any discomfort it may entail. Participants will be given every opportunity to clarify any points they do not understand and, if necessary, ask for more information. Participants are entitled to withdraw their consent to participate at any time without penalty or loss of benefits to which they are otherwise entitled.

**7.3. Participant Confidentiality**

The research staff will ensure that the participants’ anonymity is maintained. The participants will be identified by name, address, date of birth and a participants ID number. All documents will be stored securely and only accessible by trial staff and authorised personnel. The data will be anonomised as soon as it is practical to do so. Any data or samples that relate to participants that leave the study site will be identified by study number only.

**8. PUBLICATION POLICY**

The principal investigator will co-ordinate dissemination of data from this study. All publications based on this study will be reviewed by each investigator prior to submission.

**9. REFERENCES**

1. Jennings LC, Anderson TP, Benyon KA, Chua A, Laing RTR, Werno AM, Young SA, Chambers ST, Murdoch DR. Incidence and characteristics of viral community-acquired pneumonia in adults. Thorax, 2008; 63: 42-8.
2. Neil AM, Martin IR, Weir R, Anderson R, Chereshsky A, Epton MJ, Jackson R, Schousboe M, Frampton C, Hutton S, Chambers ST, Town GI. Community-acquired pneumonia: aetiology and usefulness of severity criteria on admission. Thorax, 1996; 51: 1010-6.
3. Laing R, Slater W, Coles C, Chambers S, Frampton C, Jackson R, Jennings L, Karalus N, Mills G, Murdoch D, Town I. Community-acquired pneumonia in Christchurch and Waikato 1999-2000: microbiology and epidemiology. NZMJ, 2001; 114: 488-91.
4. Chambers ST, Laing R, Murdoch D, Frampton C, Jennings L, Karalus N, Mills G, Town I. Māori have a much higher incidence of CAP and pneumococcal pneumonia than Non-Māori: findings from two hospitals. NZMJ, 2006; 119 (1234) 1-10.
5. Respiratory Disease 50+ years. Ministry of Health 2011. Tatau Kura Tangata: Health of Older Māori Chart Book 2011. Wellington, Ministry of Health.
6. Waterer GW, Rello J, Wunderink RG. Management of community-acquired pneumonia in adults. Am J Respir Crit Care Med, 2011; 183: 157-64.
7. Singanayagam A, Chalmers JD, Hill AT. Severity assessment in community-acquired pneumonia: a review. QJM. 2009;102:379-88
8. Ruuskanen O, Lahti E, Jennings LC, Murdoch DR. Viral pneumonia. Lancet, 2011; 377: 1264-75.
9. Scott G, Scott H, Turley M, Baker M. Economic cost of community-acquired pneumonia in New Zealand adults. NZMJ, 2004; 117(1196): 1-9.
10. Schmitt S. Community Acquired Pneumonia. Disease Management Project: Cleveland Clinic Center for Continuing Education 2010.
11. Madhi SA, Klugman KP, Group TVT. A role for *Streptococcus pneumoniae* in virus-associated pneumonia. Nat Med 2004; 10:811-3.
12. Madhi SA, Ludewick H, Kuwanda L, et al. Pneumococcal coinfection with human metapneumovirus. J Infect Dis 2006; 193:1236-43.
13. McCullers JA. Insights into the interaction between influenza virus and pneumococcus. Clin Microbiol *Rev* 2006; 19:571-82.
14. Talbot TR, Poehling KA, Hartert TV, Arbogast PG, Halasa NB, Edwards KM, Schaffner W, Craig AS, Griffin MR. Seasonality of invasive pneumococcal disease: temporal relation to documented influenza and respiratory syncytial virus circulation. Am J Med, 2005; 118: 285-291.
15. Ishizuka S, Yamaya M, Suzuki T, et al. Effects of rhinovirus infection on the adherence of *Streptococcus pneumoniae* to cultured human airway epithelial cells. J Infect Dis 2003; 188:1928-39.
16. Holick MF. High Prevalence of Vitamin D Inadequacy and Implications for Health. Mayo Clin Proc 2006; 81:353-73.
17. Ross AC, Taylor CL, Yaktine AL, Del Valle HB (eds). Calcium and Vitamin D. Committee to review dietary reference intakes for vitamin D and calcium food and nutrition board. IOM. National Academies Press, Washington DC, 2010.
18. Bischoff-Ferrari HA, Giovannucci E, Willett WC, Dietrich T, Dawson-Hughes B. Estimation of optimal serum concentrations of 25-hydroxyvitamin D for multiple health outcomes. Am J Clin Nutr 2006; 84:18-28.
19. Black PN, Scragg R. Relationship Between Serum 25-Hydroxyvitamin D and Pulmonary Function in the Third National Health and Nutrition Examination Survey. *Chest* 2005; 128:3792-8.
20. Forman JP, Giovannucci E, Holmes MD, et al. Plasma 25-Hydroxyvitamin D Levels and Risk of Incident Hypertension. Hypertension 2007; 49:1063-9.
21. Garland CF, Gorham ED, Mohr SB, et al. Vitamin D and prevention of breast cancer: Pooled analysis. *J Steroid Biochem Molec Biol* 2007; 103:708-11.
22. Gorham ED, Garland CF, Garland FC, et al. Optimal Vitamin D Status for Colorectal Cancer Prevention: A Quantitative Meta Analysis. *Am J Prev Med* 2007; 32:210-6.
23. Scragg R, Holdaway I, Singh V, Metcalf P, Baker J, Dryson E. Serum 25-hydroxyvitamin D3 levels decreased in impaired glucose tolerance and diabetes mellitus. Diabet Res Clin Pract*.* 1995; 27:181-8.
24. Scragg R, Jackson R, Holdaway IM, Lim T, Beaglehole R. Myocardial Infarction is Inversely Associated with Plasma 25-Hydroxyvitamin D3 Levels: A Community-Based Study. *Int J Epidemiol* 1990; 19:559-63.
25. Scragg R, Sowers M, Bell C. Serum 25-Hydroxyvitamin D, Diabetes, and Ethnicity in the Third National Health and Nutrition Examination Survey. Diabetes Care*.* 2004; 27:2813-8.
26. Scragg R, Sowers M, Bell C. Serum 25-hydroxyvitamin D, ethnicity, and blood pressure in the Third National Health and Nutrition Examination Survey. *Am J Hypertens* 2007;20:713-9.
27. Melamed ML, Michos ED, Post W, Astor B. 25-Hydroxyvitamin D levels and the risk of mortality in the general population. Arch Intern Med*.* 2008; 168:1629-37.
28. Lips P. Worldwide status of vitamin D nutrition. J Steroid Biochem Mol Biol, 2010; 121: 297-300.
29. Ministry of Health 2012. Vitamin D status of New Zealand Adults: Findings from the 2008/09 New Zealand Adult Nutritional Survey. Wellington: Ministry of Health.
30. Livesey J, Elder P, Ellis MJ, McKenzie R, Liley B, Florkowski C. Seasonal variation in vitamin D levels in the Canterbury, New Zealand population in relation to available UV radiation. *NZ Med J.* 2007; 120:U2733.
31. Salmon PJ, Chan WC, Griffin J, McKenzie R, Rademaker M. Extremely high levels of melanoma in Tauranga, New Zealand: Possible causes and comparisons with Australia and the northern hemisphere. Australas J Dermatol 2007; 48:208-16.
32. Nowson CA, Margerison C. Vitamin D intake and vitamin D status of Australians. *Med J Aust* 2002; 177:149-52.
33. Heaney RP, Davies KM, Chen TC, Holick MF, Barger-Lux MJ. Human serum 25-hydroxycholecalciferol response to extended oral dosing with cholecalciferol. Am J Clin Nutr 2003; 77:204-10.
34. Barger-Lux MJ, Heaney RP, Dowell S, Chen TC, Holick MF. Vitamin D and its Major Metabolites: Serum Levels after Graded Oral Dosing in Healthy Men. *Osteoporosis Int.* 1998; 8:222-30.
35. Vieth R, Chan P-CR, MacFarlane GD. Efficacy and safety of vitamin D3 intake exceeding the lowest observed adverse effect level. *Am J Clin Nutr* 2001;73:288-94.
36. Tripkovic L, lambert H, Hart K, Smith CP, Bucca G, Penson S, Chope G, Hypönen E, Berry J, Vieth R, Lanham-New S. Comparison of vitamin D_2_ and vitamin D_3_ supplementation in raising serum 25-hydroxyvitamin D status: a systematic review and meta-analysis. Am J Clin Nutr, 2012; 1357-64.
37. Armas LA, Hollis BW, Heaney RP. Vitamin D_2_ is much less effective than vitamin D_3_ in humans. J Clin Endocrinol Metab, 2004; 89: 5387-5391.
38. Romagnoli E, Mascia ML, Cipriani C, Fassino V, Mazzei F, D’Erasmo E, Carnevale V, Scillitani A, Minisola S. Short and long-term variations in serum calciotropic hormones after a single very large dose of ergocalciferol (vitamin D_2_) or cholecalciferol (vitamin D_3_) in the elderly. J Clin Endocrinol Met, 2008; 93: 3015-20
39. Ilahi M, Armas LAG, Heaney RP. Pharmacokinetics of a single, large dose of cholecalciferol. Am J Clin Nutr, 2008; 87: 688-91.
40. White JH. Vitamin D Signaling, Infectious Diseases, and Regulation of Innate Immunity. Infect Immun 2008; 76:3837-43.
41. Ganz T. Defensins: antimicrobial peptides of innate immunity. Nat Rev Immunol 2003; 3:710-20.
42. Zasloff M. Fighting infections with vitamin D. Nat Med 2006; 12:388-90.
43. Liu PT, Stenger S, Li H, et al. Toll-Like Receptor Triggering of a Vitamin D-Mediated Human Antimicrobial Response. Science 2006; 311:1770-3.
44. Dürr UHN, Sudheendra US, Ramamoorthy A. LL-37, the only human member of the cathelicidin family of antimicrobial peptides. Biochim Biophys Acta (BBA) - Biomemb 2006; 1758:1408-25.
45. Baeke F, Takiishi T, Korf H, Gysemans C, Mathieu C. Vitamin D: modulator of the immune system. Curr Opin Pharmacol, 2010; 10: 482-96.
46. Martineau AR, Honecker FU, Wilkinson RJ, Griffiths CJ. Vitamin D in the treatment of pulmonary tuberculosis. J Steroid Biochem Molec Biol 2007; 103:793-8.
47. Wilkinson RJ, Llewelyn M, Toossi Z, et al. Influence of vitamin D deficiency and vitamin D receptor polymorphisms on tuberculosis among Gujarati Asians in west London: a case-control study. Lancet*.* 2000; 355:618-21.
48. Ustianowski A, Shaffer R, Collin S, Wilkinson RJ, Davidson RN. Prevalence and associations of vitamin D deficiency in foreign-born persons with tuberculosis in London. J Infect*.* 2005; 50:432-7.
49. Wejse C, Olesen R, Rabna P, et al. Serum 25-hydroxyvitamin D in a West African population of tuberculosis patients and unmatched healthy controls. Am J Clin Nutr 2007; 86:1376-83.
50. Wilbur AK, Salter Kubatko L, Hurtado AM, Hill KR, Stone AC. Vitamin D receptor gene polymorphisms and susceptibility M. tuberculosis in Native Paraguayans. Tuberculosis 2007; 87:329-37.
51. Stroder J, Kasal P. Phagocytosis in vitamin D deficient rickets. Klin Wochenschr 1970; 48:383-4.
52. Banajeh SM, Al-Sunbali NN, Al-Sanahani SH. Clinical characteristics and outcome of children aged under 5 years hospitalized with severe pneumonia in Yemen. Annal Trop Paediat 1997; 17:321.
53. Beser E, Cakmakci T. Factors affecting the morbidity of vitamin D deficiency rickets and primary protection. *East Afr Med J* 1994; 71:358-62.
54. El-Radhi AS, Majeed M, Mansor N, Ibrahim M. High incidence of rickets in children with wheezy bronchitis in a developing country. J Roy Soc Med 1982; 75:884-7.
55. Mariam TW, Sterky G. Severe rickets in infancy and childhood in Ethiopia. J Pediatr 1973; 82:876-8.
56. Muhe L, Lulseged S, Mason KE, Simoes EAF. Case-control study of the role of nutritional rickets in the risk of developing pneumonia in Ethiopian children. Lancet 1997; 349:1801-4.
57. Najada AS, Habashneh MS, Khader M. The Frequency of Nutritional Rickets among Hospitalized Infants and its Relation to Respiratory Diseases. J Trop Pediatr 2004; 50:364-8.
58. Patwari A, Nabi G, Nadroo AM, Singh D, Manhas RS. Pulmonary changes in rickets in children. Ind Pediatr 1979; 16:413-5.
59. Siddiqui TS, Rai MI. Presentation and predisposing factors of nutritional rickets in children of Hazara Division. J Ayub Med Coll *Abbottabad.* 2005; 17:29-32.
60. Wayse V, Yousafzai A, Mogale K, Filteau S. Association of subclinical vitamin D deficiency with severe acute lower respiratory infection in Indian children under 5 y. Eur J Clin Nutr 2004; 58:563-7.
61. Holick MF. Resurrection of vitamin D deficiency and rickets. J Clin Invest 2006; 116:2062-72.
62. Gigineishvili GR, Il'in NI, Suzdal'nitskii RS, Levando VA. [The use of UV irradiation to correct the immune system and decrease morbidity in athletes]. Vopr Kurortol Fizioter Lech Fiz Kult 1990; 3:30-3.
63. Termorshuizen F, Wijga A, Gerritsen J, Neijens HJ, van Loveren H. Exposure to solar ultraviolet radiation and respiratory tract symptoms in 1-year-old children. Photodermatol Photoimmunol *Photomed* 2004; 20:270-1.
64. Cannell J, Zasloff M, Garland C, Scragg R, Giovannucci E. On the epidemiology of influenza. Virol J*.* 2008; 5:29.
65. Cannell JJ, Vieth R, Umhau JC, et al. Epidemic influenza and vitamin D. Epidemiol Infect 2006; 134:1129-40.
66. Karatekin G, Kaya A, Salihoğlu Ö, Balci H, Nuhoğlu A. Association of subclinical vitamin D deficiency in newborns with acute lower respiratory infection and their mothers. Eur J Clin Nutr 2009; 63:473-7.
67. Laaksi I, Ruohola J-P, Tuohimaa P, et al. An association of serum vitamin D concentrations < 40 nmol/L with acute respiratory tract infection in young Finnish men. Am J Clin Nutr 2007; 86:714-7.
68. Roth DE, Jones AB, Prosser C, Robinson JL, Vohra S. Vitamin D status is not associated with the risk of hospitalization for acute bronchiolitis in early childhood. Eur J Clin Nutr 2009; 63:297-9.
69. Ginde AA, Mansbach JM, Camargo CA. Association between serum 25-hydroxyvitamin D level and upper respiratory tract infections in the Third National Health and Nutrition Examination Survey. Arch Intern Med 2009; 169:384-90.
70. Leow L, simpson T, Cursons R, Karalus N, Hancox RJ. Vitamin D, innate immunity and outcomes in community acquired pneumonia. Respirology, 2011; 16: 611-6.
71. Aloia JF, Li-Ng M. Re: epidemic influenza and vitamin D. Epidemiol Infect 2007; 135:1095-6.
72. Avenell A, Cook JA, MacLennan GS, MacPherson GC. Vitamin D supplementation to prevent infections: a sub-study of a randomised placebo-controlled trial in older people (RECORD trial, ISRCTN 51647438). Age Ageing*.* 2007; 36:574-7.
73. Li-Ng M, Aloia JF, Pollack S, et al. A randomized controlled trial of vitamin D3 supplementation for the prevention of symptomatic upper respiratory tract infections. Epidemiol Infect 2009; 137:1396-404.
74. Murdoch DR, Slow S, Chambers ST, Jennings LC, Stewart AW, Priest PC, Florkowski CM, Livesey JH, Camargo C, Scragg R. Effect of vitamin D3 supplementation on upper respiratory infections in healthy adults: The VIDARIS randomised controlled trial. JAMA, 2012; 308: 1333-1339.
75. Camargo Jr CA, Ganmaa D, Frazier AL, Kirchberg FF, Stuart JJ, Kleinman SK, Sumberzul N, Rich-Edwards JW. Randomized trial of vitamin D supplementation and risk of acute respiratory infection in Mongolia. Pediatrics, 2012; 130: e561-67.
76. Manaseki-Holland S, Maroof Z, Bruce J, Mughal MZ, Masher MI, Bhutta ZA, Walraven G, Chandramohan D. Effect on the incidence of pneumonia of vitamin D supplementation by quarterly bolus dose to infants in Kabul: a randomised controlled superiority trial. Lancet, 2012; 379: 1419-27.
77. Martineau AR, Timms PM, Bothamley GH, Hanifa Y, Islam K, Claxton AP, Packe GE, Moore-Gillon JC, Darmalingam M, Davidson RN, Milburn HJ, Baker LV, Barker RD, Woodward NJ, Venton TR, Barnes KE, Mullett CJ, Coussens AK, Rutterford CM, Mein CA, Davis GR, Wilkinson RJ, Nikolayevskyy V, Drobniewski FA, Eldridge SM, Griffiths CJ. High dose vitamin D_3_ during intensive-phase antimicrobial treatment of pulmonary tuberculosis: a double-blind randomised controlled trial. Lancet, 2011; 377: 242-50.
78. Coussens AK, Wilkinson RJ, Hanifa Y, Nikolayevskyy V, Elkington PT, Islam K, Timms PM, Venton TR, Bothamley GH, Packe GE, Darmalingam M, Davidson RN, Milburn HJ, Baker LV, Barker RD, Mein CA, Bhaw-Rosun L, Nuamah R, Young DB, Drobniewski FA, Griffiths CJ, Martineau AR. Vitamin D accelerates resolution of inflammatory responses during tuberculosis treatment. PNAS, 2012; 38: 15449-54.
79. Salahuddin N, Ali F, Hasan Z, Rao N, Ageel M, Mahmood F. Vitamin D accelerates clinical recovery from tuberculosis: results of the SUCCINCT study [supplementary cholecalciferol in recovery from tuberculosis]. A randomised, placebo-controlled, clinical trial of vitamin D supplementation in patients with pulmonary tuberculosis. BMC Infect Dis, 2013; 13: 22.
80. Wejse C, Gomes VF, Rabna P, Gustafson P, Aaby P, Lisse IM, Andersen PL, Glerup H, Sodemann M. Vitamin D as supplementary treatment for tuberculosis. Am J Respir Crit Care Med, 2009; 179: 843-50.
81. Soilu-Hänninen M, Aivo J, Lindström, Elovaara I, sumelahti M-L, Färkkilä M, Tienari P, Atula S, Sarasoja T, Herrala L, Keskinarkaus I, Kruger J, Kallio T, Rocca MA, Filippi M. A randomised, double blind, placebo controlled trial with vitamin D_3_ as an add on treatment to interferon β-1b in patients with multiple sclerosis.J Neurol Neurosurg Psychiatry, 2012; 83: 565-71.
82. Shaygannejad V, Janghorbani M, Ashtari F, Dehghan H. Effects of adjunct low-dose vitamin D on relapsing-remitting multiple sclerosis progression: preliminary findings of a randomized placebo-controlled trial. Multiple Sclerosis Int, 2012; doi:10.1155/2012/452541.
83. Grossmann RE, Zughaier SM, Kumari M, Seydafkan S, Lyles RH, Liu S, Sueblinvong V, Schechter MS, Stecenko AA, Ziegler TR, Tangpricha V. Pilot study of vitamin D supplementation in adults with cystic fibrosis pulmonary exacerbation. A randomized controlled trial. Dermato-Endocrinology, 2012; 4: 191-197.
84. Choudhary N, Gupta P. Vitamin D supplementation for severe pneumonia-a randomized controlled trial. Indian Pediatrics, 2012; 49: 449-54.
85. Manaseki-Holland S, Qader G, Masher MI, Bruce J, Mughal MZ, Chandramohan D, Walraven G. Effects of vitamin D supplementation to children diagnosed with pneumonia in Kabul: a randomised controlled trial. Trop Med Int Health, 2010; 15: 1148-55.
86. Valdivielso JM, Fernandez E. Vitamin D receptor polymorphisms and diseases. Clin Chim Acta 2006; 371:1-12.
87. Barrett B, Brown R, Mundt M, et al. The Wisconsin Upper Respiratory Symptom Survey is responsive, reliable, and valid. J Clin Epidemiol 2005; 58:609-17.
88. Wang TJ, Zhang F, Richards JB, Kestenbaum B, van Meurs JB, Berry D, et al. Common genetic determinants of vitamin D insufficiency: a genome-wide association study. The Lancet 2010; 376:180-188.
89. Ahn J, Yu K, Stolzenberg-Solomon R, Simon KC, McCullough ML, Gallicchio L, et al. Genome-wide association study of circulating vitamin D levels. Hum Mol Genet 2010; 19:2739-45.
90. Dastani Z, Goltzman D, Wang T, Fu L, Kiel DP, Cole D, Richards B. Influence of genetic Variants on response to vitamin D administration (conference proceedings abstract). American Human Genetics Society Meeting, Montreal 2011.
91. Sanders KM, Stuart AL, Williamson EJ, Simpson JA, Kotowicz MA, Young D, Nicholson GC. Annual high-dose oral vitamin D and falls and fractures in older women. A randomized controlled trial. JAMA, 2010; 303: 1815-22.
92. Bacon C, Gamble G, Horne A, Scott M, Reid I. High-dose vitamin D_3_ supplementation in the elderly. Osteoporos Int, 2009; 20: 1407-15.
93. Amrein K, sourij H, Wagner G, Holl A, Pieber TR, smolle KH, Stojakovic T, Schned C, Dobnig H. Short-term effects of high-dose oral vitamin D3 in critically ill vitamin D deficient patients: a randomized, double-blind, placebo-controlled pilot study. Critical Care, 2011; 15: R104.
94. Jones G. Pharmacokinetics of vitamin D toxicity. *Am J Clin Nutr* 2008;88:582-6.
